# Supplementary material for: SMPDL3b modulates insulin receptor signaling in diabetic kidney disease
Source: Nat Commun. 2019 Jun 19;10:2692. doi: 10.1038/s41467-019-10584-4 (PMC6584700; doi:10.1038/s41467-019-10584-4)
Supplement: Supplementary file 1 — Supplementary Information [file 41467_2019_10584_MOESM1_ESM.pdf]

Supplementary Information

SMPDL3b Modulates Insulin Receptor Signaling in Diabetic Kidney Disease

Mitrofanova et al.

Supplementary Figures

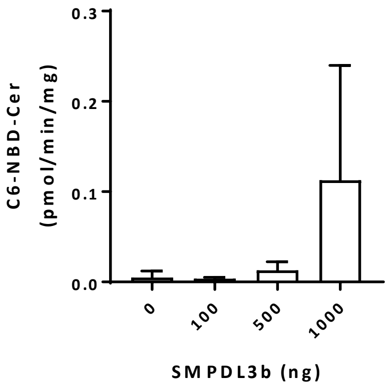

Supplementary Fig. 1. SMPDL3b shows C1P phosphatase activity in transfected HEK293 cells. C1P *in vitro* assay using 50 μM C6-NBD-C1P in presence of increased concentration of purified SMPDL3b protein. n=2 in duplicate. P=1.372, F=0.37, One-Way ANOVA. Error bars represent standard deviation (S.D.).

a

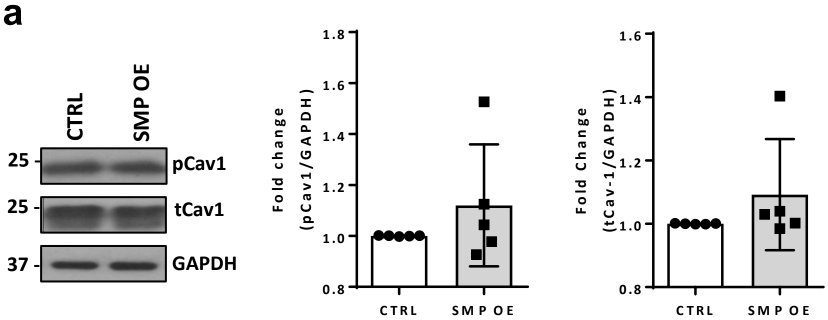

b

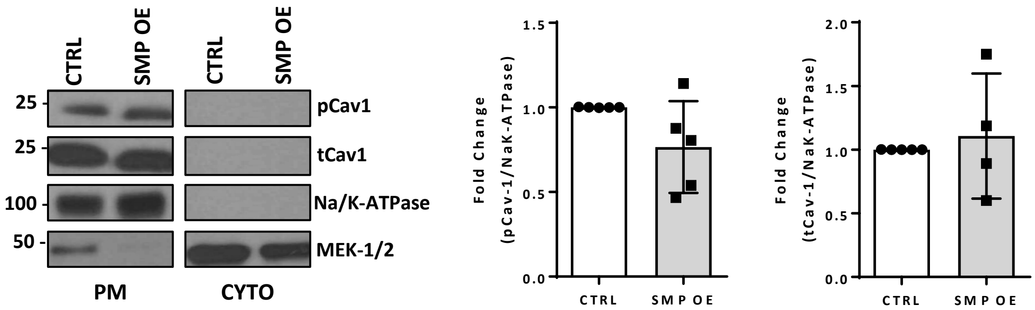

Supplementary Fig. 2. SMPDL3b overexpression in human podocytes makes no changes in expression of caveolin-1. **(a)** Western blot and bar graph analyses of phosphorylated (pCav1) and total (tCav1) caveolin-1 expression in lysates of control (CTRL) and SMPDL3b overexpression (SMP OE) podocytes. n=5, P=0.295 for pCav1, P=0.275 for tCav1, two-tailed t-test. **(b)** Western blot of phosphorylated (pCav1) and total (tCav1) caveolin-1 in plasma membrane (PM) or cytosolic fraction (CYTO) in CTRL or SMP OE podocytes. A graph showing no changes in pCav1 and tCav1 at the plasma membrane in CTRL and SMP OE podocytes. n=5; P=0.09 for pCav1, P=0.634, two-tailed t-test. Error bars represent standard deviation (S.D.).

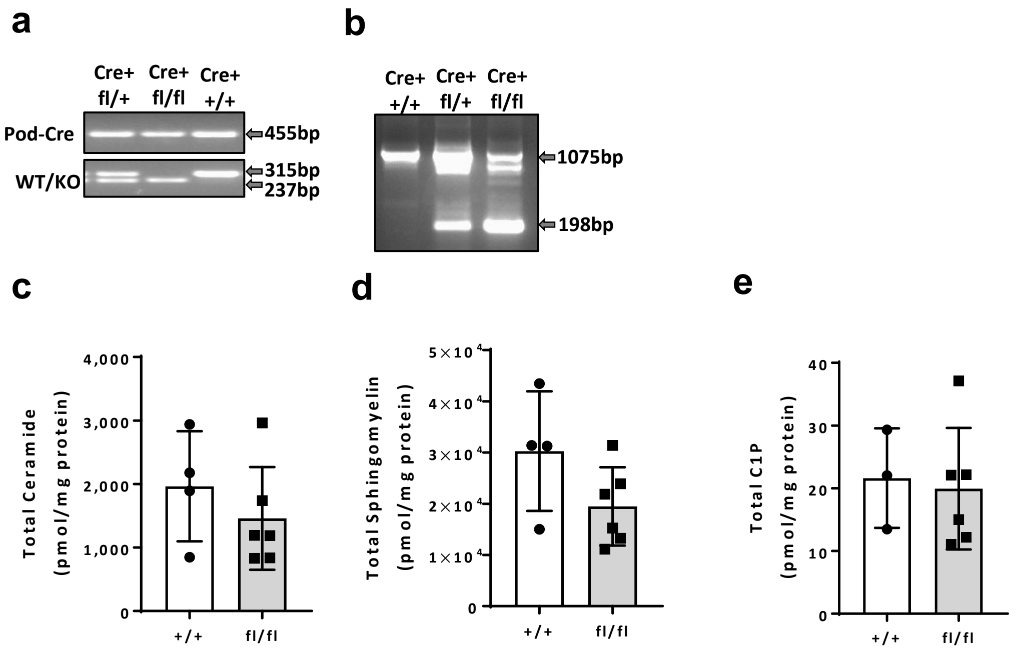

Supplementary Fig. 3. Genotyping PCR example of the podocyte-specific *Smpdl3b* deficient mice, detection of Cre-LoxP mediated deletion of *Smpdl3b* and lipidomic analysis of kidney cortexes. **(a)** PCR on genomic DNA isolated from tails biopsy showing amplification products of 315bp for wild-type allele in Cre+;+/+ mice, 237bp for *Smpdl3b* floxed allele in Cre+;fl/fl and both bands in heterozygous, Cre+;fl/+ mice (WT/KO). A 455bp PCR product is detected in mice expressing the Podocin-Cre transgene (Pod-Cre). **(b)** PCR on genomic DNA isolated from glomeruli showing a specific amplification product of 198bp which is only presented in Cre+;fl/+ and Cre+;fl/fl mice. **(c, d, e)** LC-MS analysis of total ceramide, P=0.37 **(c)**; total sphingomyelin, P=0.11 **(d)** and total ceramide-1-phosphate (C1P), P=0.80 **(e)** content in kidney cortexes of Cre+;+/+ (n=4) and Cre+;fl/fl (n=6) mice. Two-tailed t-test. Error bars represent standard deviation (S.D.).

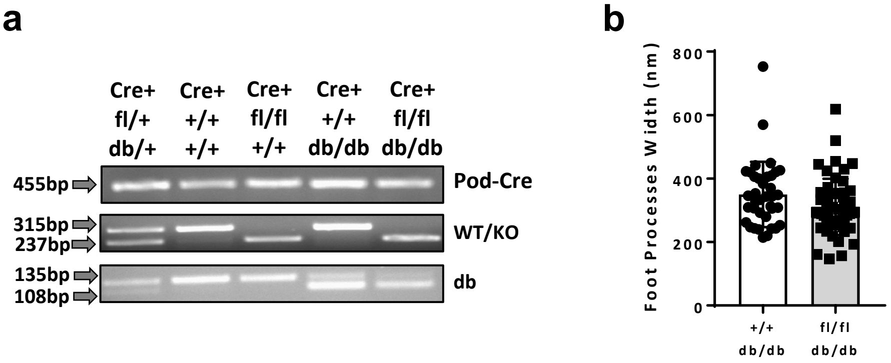

Supplementary Fig. 4. Genotyping example of diabetic mice with podocyte-specific *Smpdl3b* deficiency. **(a)** PCR on genomic DNA isolated from tails biopsy showing amplification products of 315bp for control mice (Cre+;+/+), 237bp for *Smpdl3b* knockout (Cre+;fl/fl) and both bands in heterozygous (Cre+;fl/+) mice (WT/KO). A 455bp PCR product is detected in mice expressing the Cre-recombinase transgene (Pod-Cre). To detect the *Lepr*^{db} mutation in diabetic mice, db primers were used. Only the lower band (108 bp) is detected in homozygous db/db mice, while two bands, 135 bp and 108 bp, can be detected in heterozygous db/+ mice. In wild type mice only the upper band (135 bp) was detected. **(b)** Foot processes width (nm) in diabetic control mice (+/+; db/db) and diabetic mice with podocyte-specific *Smpdl3b* deletion (fl/fl;db/db). P=0.055, two-tailed t-test. Error bars represent standard deviation (S.D.).

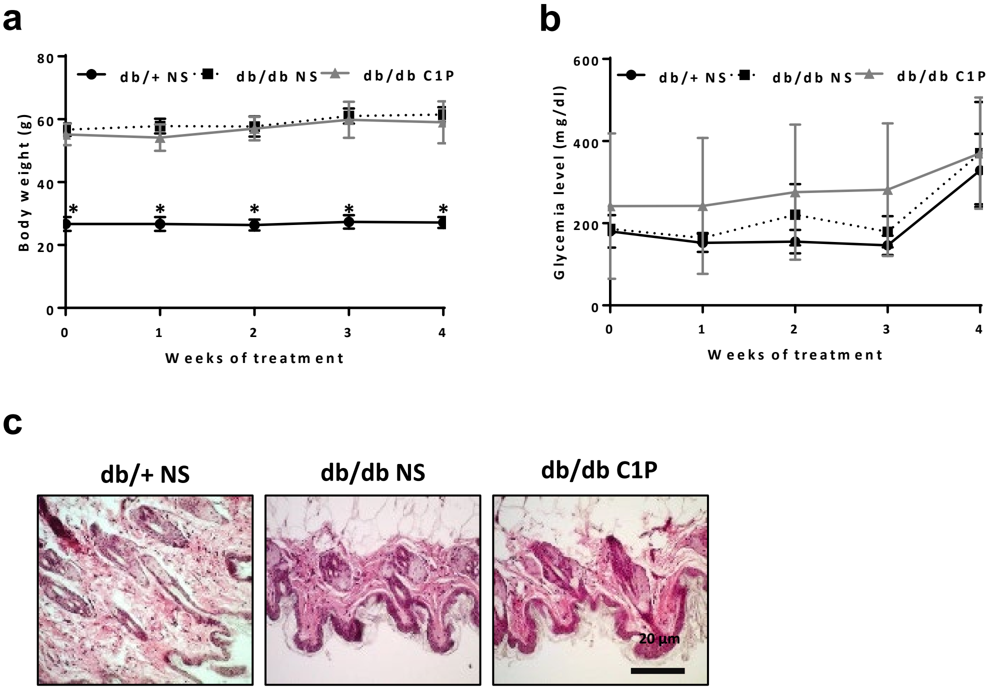

Supplementary Fig. 5. Phenotype of db/db mice treated with exogenous ceramide-1-phosphate C16:0 (C1P). For exogenous C1P administration *in vivo* three groups of 12 weeks-old mice were utilized: 1) control heterozygous mice intraperitonially injected with 0.9% normal saline (db/+ NS; n=6), 2) diabetic mice intraperitonially injected with 0.9% normal saline (db/db NS; n=6), 3) diabetic mice intraperitonially injected with 30mg/kg C1P for 28 days (db/db C1P; n=6). **(a)** A graph showing body weight changes in db/+ NS, db/db NS and db/db C1P mice during the study.

(b) A graph showing random glycemia level changes in db/+ NS, db/db NS and db/db C1P mice during the study. **(c)** Representative hematoxylin-eosin staining of skin sections (4 μm) from db/+ mice, db/db NS mice and db/db C1P mice. No toxic effect of C1P at the injection site was found. Error bars represent standard deviation (S.D.).

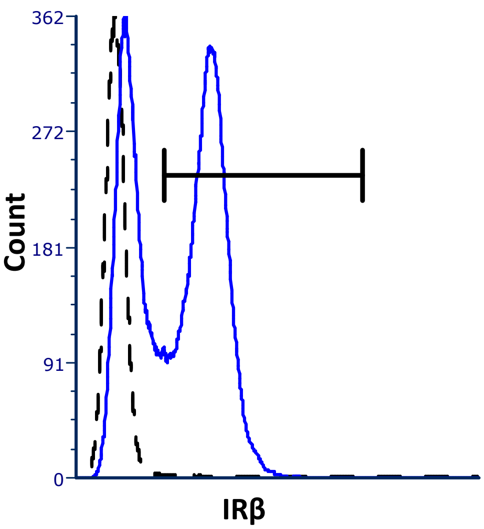

Supplementary Fig. 6. Flow cytometry gating strategy used for insulin receptor expression analysis in control and SMPDL3b overexpressing podocytes. Blue line is a positive control; black dash line is a negative control.

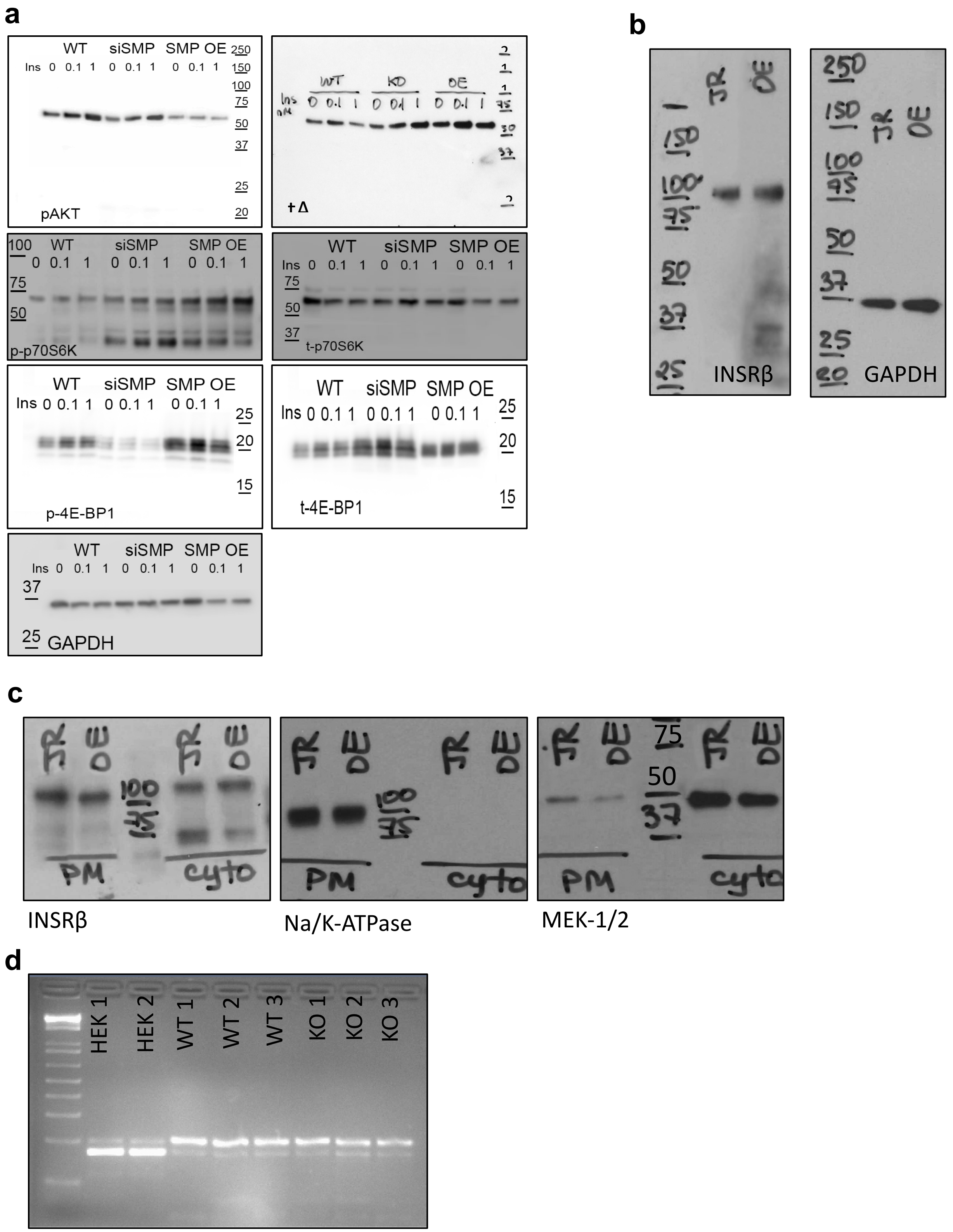

Supplementary Fig. 7. Uncropped Western blot pictures related to Fig. 2. **(a)** Representative Western blot image of main downstream targets of the insulin receptor signaling in control (CTRL), SMPDL3b knockdown (siSMP) and SMPDL3b overexpressing (SMP OE) podocytes exposed to

increasing concentration of insulin (0, 0.1, 1 nM): phosphorylated (pAKT) and total (tAKT) AKT, phosphorylated (p-p70S6K) and total (t-p70S6K) p70S6K, phosphorylated (p-4EB-P1) and total (t-4EB-P1) 4EB-P1. GAPDH served as a loading control. **(b)** Representative Western blot analysis of insulin receptor (IR) expression in total lysates of CTRL and SMP OE podocytes. **(c)** Representative Western blot of IR localization at the plasma membrane (PM) in CTRL and SMP OE podocytes. Na/K-ATPase was used as a marker for the PM fraction and MEK-1/2 was used as a marker of the cytosolic fraction (CYTO). **(d)** Representative PCR of the insulin receptor isoform A and isoform B expression in HEK293, control (WT) and SMP OE (OE) human podocytes.

a

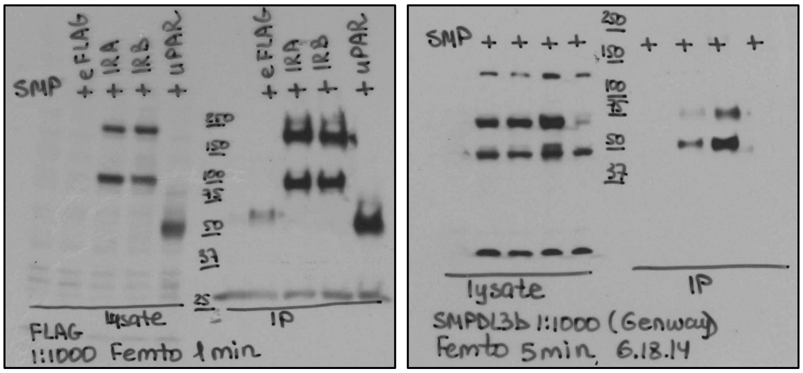

b

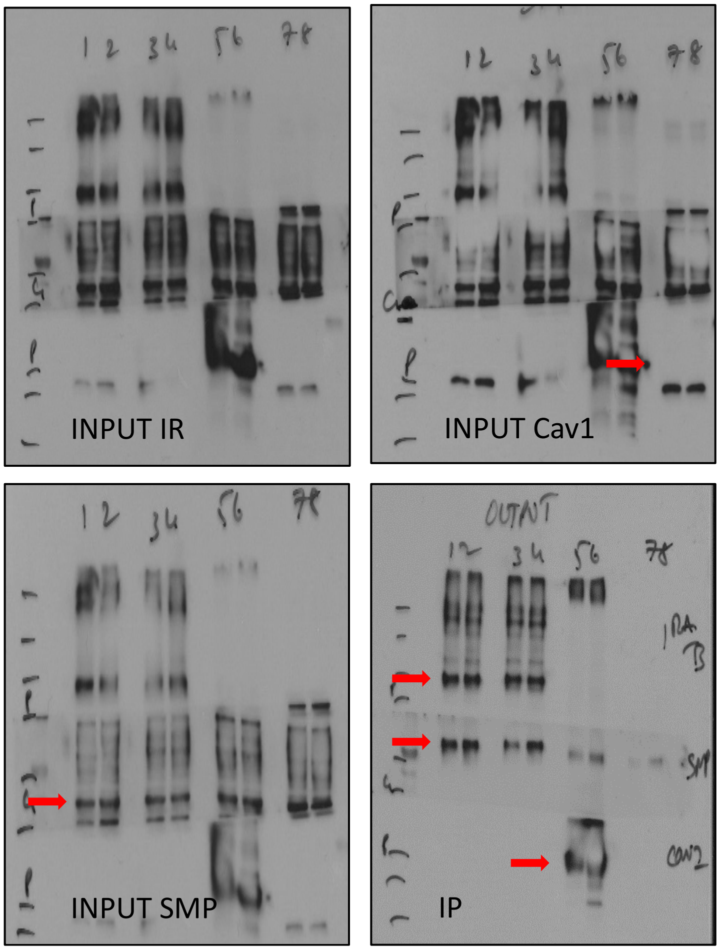

c

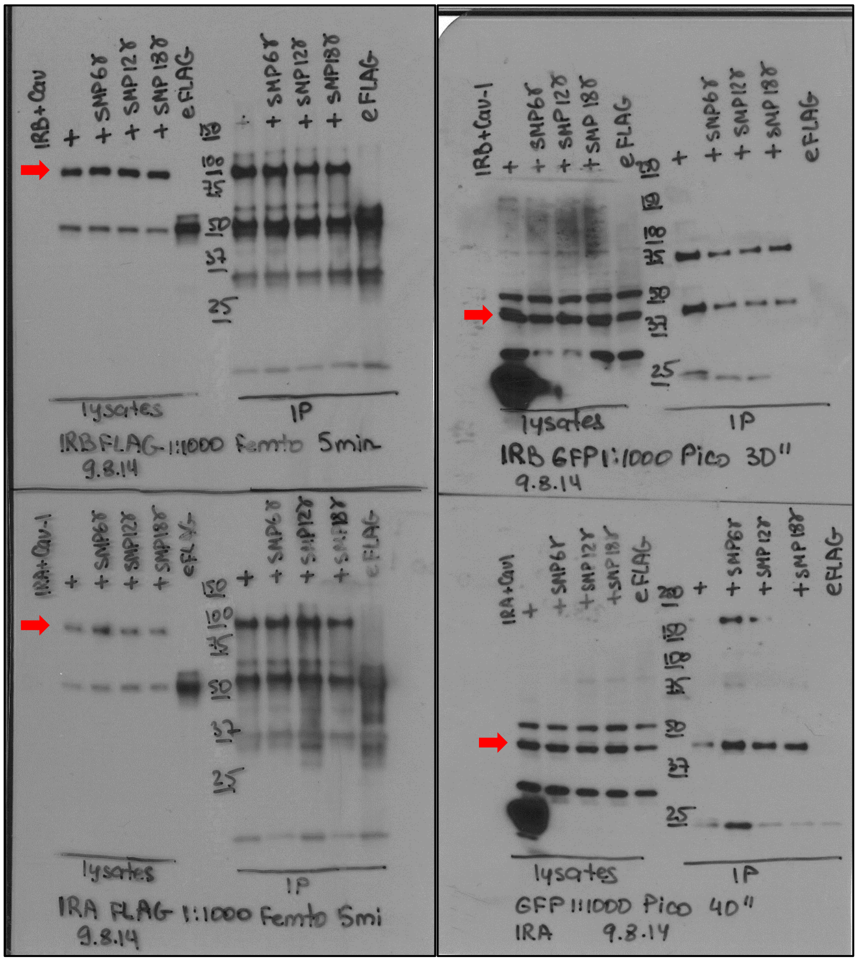
lysates
IP
IRB FLAG 1:1000 Femto 5min
9.8.14
IRB GFP 1:1000 Pico 30"
9.8.14
IRA FLAG 1:1000 Femto 5mi
9.8.14
GFP 1:1000 Pico 40"
IRA 9.8.14

d

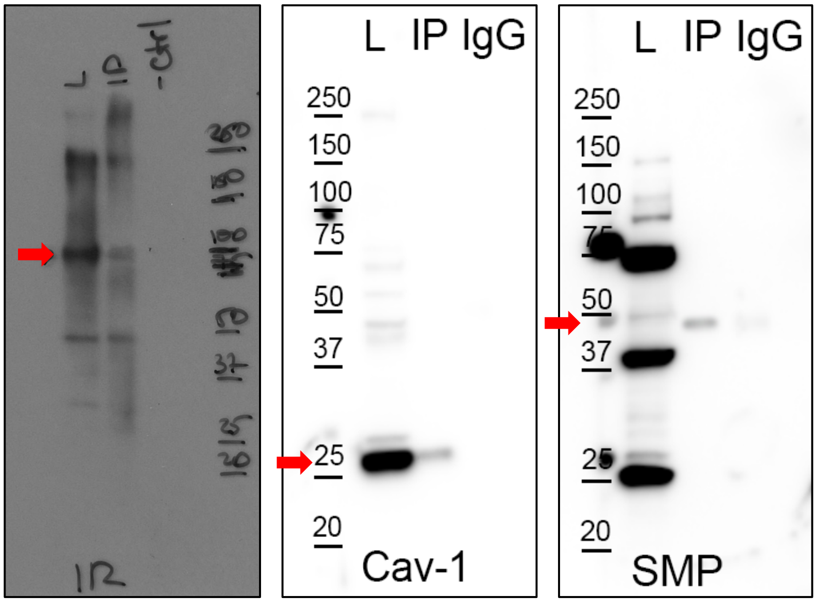
L
IP
-Ctrl
IR
L IP IgG
250
150
100
75
50
37
25
20
Cav-1
SMP

e

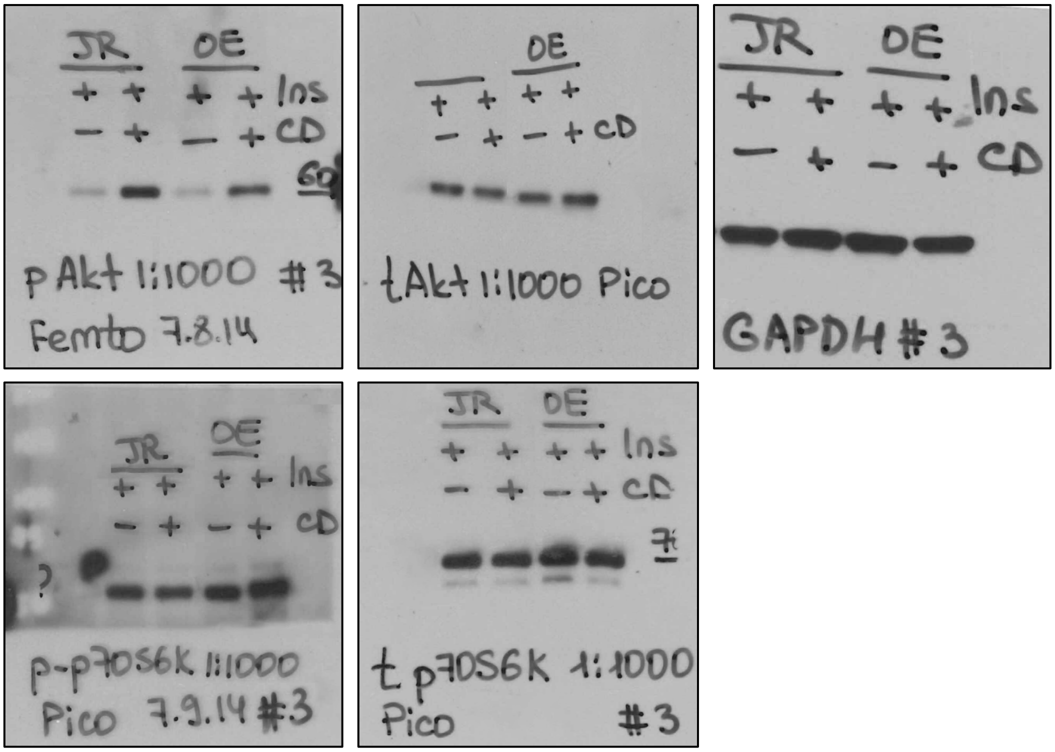
JR
OE
Ins
CD
pAkt 1:1000 #3
Femto 7.8.14
tAkt 1:1000 Pico
GAPDH #3
p-p70S6K 1:1000
Pico 7.9.14 #3
t p70S6K 1:1000
Pico #3

f

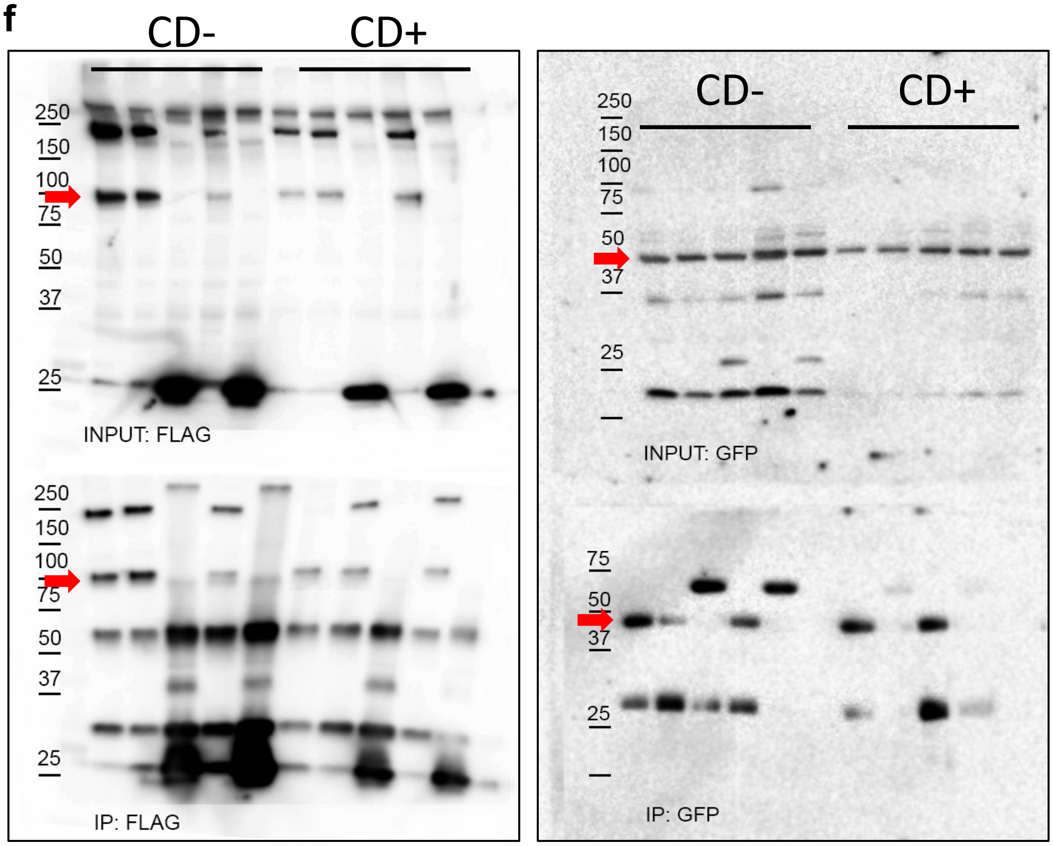
CD-
CD+
250
150
100
75
50
37
25
INPUT: FLAG
IP: FLAG
INPUT: GFP
IP: GFP

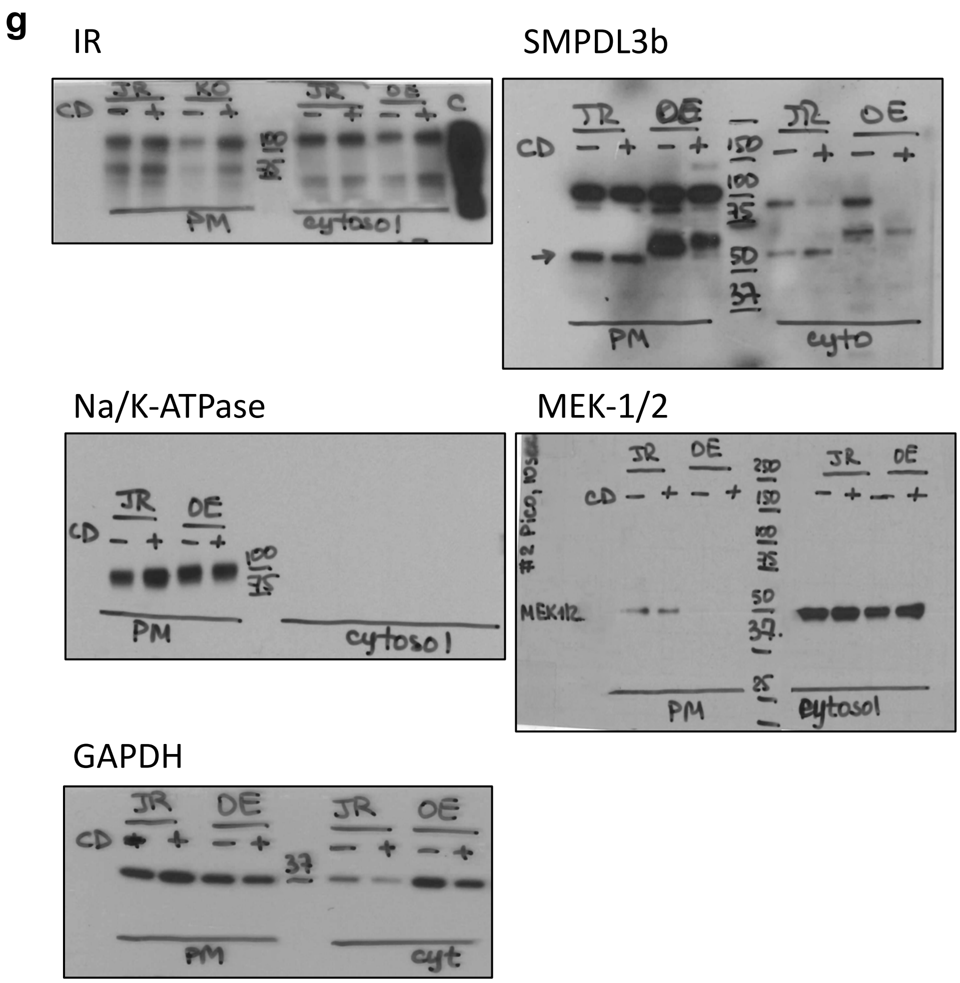

Supplementary Fig. 8. Uncropped Western blot pictures related to Fig. 3. **(a)** Representative Western blot of SMPDL3b interaction with both insulin receptor (IR) isoforms. **(b)** Representative Western blot of wildtype SMPDL3b and mutant (H135A) SMPDL3b interaction with IRA, IRB and caveolin-1. **(c)** Representative Western blot of interaction between caveolin-1 and insulin receptor isoforms in the presence of SMPDL3b overexpression. **(d)** Representative Western blot of endogenous IP experiments using glomeruli isolated from five C57BL/6 mice indicate that SMPDL3b immunoprecipitates insulin receptor (IR) and caveolin-1 (cav-1). L – lysate; IP – immunoprecipitated; IgG served as a negative control. **(e)** Representative Western blot of phosphorylated AKT (pAKT), phosphorykated p70S6K (p-p70S6K), total AKT (tAKT), total p70S6K (t-p70S6K) and GAPDH in control (CTRL) and SMPDL3b overexpressing (SMP OE) podocytes pre-treated with 5 mM methyl-β-cyclodextrin (CD) and stimulated with 1 nM insulin. **(f)** Representative Western blot of IP experiments performed in HEK293 cells co-transfected with human hIRA-FLAG, hIRB-FLAG, hCav1-GFP and hSMPDL3b-HA in presence or absence of CD. **(g)** Representative Western blot of the insulin receptor (IR) and SMPDL3b localization at the plasma membrane (PM) in CTRL and SMP OE podocytes exposed to CD and their loading controls (Na/K-ATPAase is marker of PM, MEK-1/2 is a marker of cytosolic fraction, GAPDH is found in both fractions).

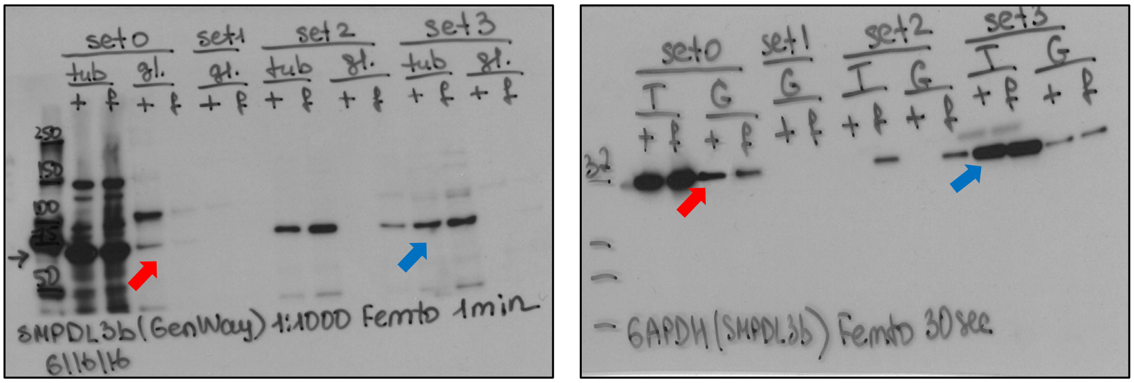

Supplementary Fig. 9. Uncropped Western blot pictures related to Fig. 4. Representative Western blot of SMPDL3b and GAPDH expression in glomeruli (red arrow) and tubules (blue arrow) pooled from 2-5 mice per group.

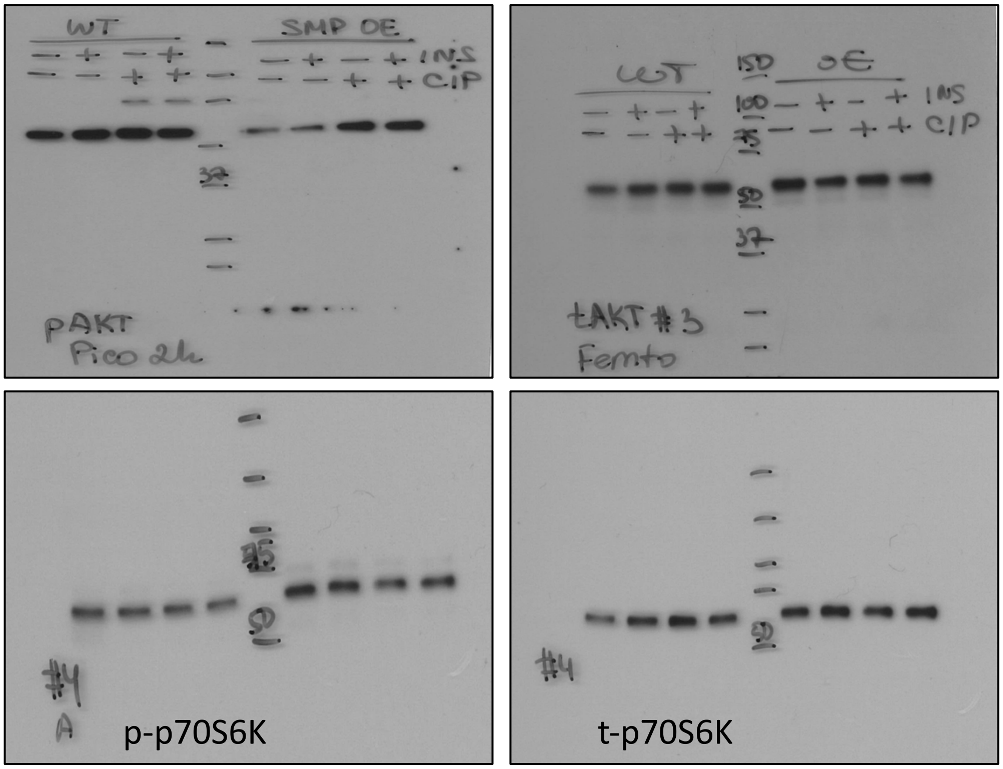

Supplementary Fig. 10. Uncropped Western blot pictures related to Fig. 6. Representative Western blot of phosphorylated AKT (pAKT), total AKT (tAKT), phosphorylated p70S6 kinase (p-p70S6K), and total p70S6 kinase (t-p70S6K) in control (WT) and SMPDL3b overexpressing (SMP OE) human podocytes pre-treated with synthetic C1P (100 μM) and treated with insulin (1nM).

Supplementary Tables

Supplementary Table 1. List of primers used in the study.

Primer name	Sense 5' → 3'	Antisense 5' → 3'	Application
FRT	AAG GCG CAT AAC GAT ACC AC	ACC AGC TCT GTT CAG CCA GT	Genotyping
PodCre	CGC ACT TCA GTT ACT TCA GGT CCT	GCT TAT GCC TGA TGT TGA TGA TGC	Genotyping
WT	GCA CCT GGT TCA GCA CTT TG	CAG CTC TGT TCA GCC AGT GA	Genotyping
KO	TGG CGC AAC GCA ATT AAT GA	AGA ACG TCA TGG GTT CAT CC	Genotyping
db	AGA ACG GAC ACT CTT TGA AGT CTC	CAT TCA AAC CAT AGT TTA GGT TTG TGT	Genotyping
5FRT/ Smp_del	AGG CGC ATA ACG ATA CCA CGA T	CCC CCT GAA CCT GAA ACA TA	PCR on genomic DNA from glomeruli
FRT_del	AAG GCG CAT AAC GAT ACC AC	N/A	PCR on genomic DNA from glomeruli
IR isoforms exon 10	TGA GGA TTA CCT GCA CAA CG	CTT CTC AAA AGG CCT GTG CT	mRNA expression level

Supplementary Table 2. Serological analysis of diabetic db/db mice and diabetic db/db mice with podocyte-specific *Smpdl3b* deficiency.

	+/+;db/db	fl/fl;db/db	P-value*
**BUN (mg/dl)**	21.9±2.7	22.5±5.5	0.778
**Serum Creatinine (mg/dl)**	0.8±0.1	0.8±0.1	0.466
**Glycemia (mg/dl)**	337.6±84.4	265.3±71.3	0.067
**ALT (u/l)**	495.9±339.0	411.1±286.7	0.634
**AST (u/l)**	351.3±192.6	400.3±209.5	0.670
**Total cholesterol (mg/dl)**	210.6±32.2	170.5±58.3	0.111
**Triglycerides (mg/dl)**	123.0±79.6	73.4±24.5	0.114

ALT – alanine aminotransferase; AST – aspartate aminotransferase. No significant differences were found between groups of mice. *Two-tailed t-test.

Supplementary Table 3. Serological analysis of db/db mice treated with ceramide-1-phosphate (C1P) or 0.9% normal saline solution.

	db/db NS	db/db C1P	P-value*
**BUN (mg/dl)**	15.6±0.5	16.7±1.1	0.439
**Serum creatinine (mg/dl)**	0.6±0.1	0.5±0.1	0.055
**ALT (u/l)**	407.8±41.3	281.0±69.6	0.172
**AST (u/l)**	379.8±50.0	287.0±41.4	0.183
**Total cholesterol (mg/dl)**	227.4±15.5	199.3±26.5	0.410
**Triglycerides (mg/dl)**	58.4±5.3	80.5±23.4	0.423

ALT – alanine aminotransferase; AST – aspartate aminotransferase. No significant differences were found between groups of mice. *Two-tailed t-test.
