# Supplementary material for: SMPDL3b modulates insulin receptor signaling in diabetic kidney disease
Source: Nat Commun. 2019 Jun 19;10:2692. doi: 10.1038/s41467-019-10584-4 (PMC6584700; doi:10.1038/s41467-019-10584-4)
Supplement: Supplementary file 3 — Description of Additional Supplementary Files [file 41467_2019_10584_MOESM3_ESM.docx]

**Description of Additional Supplementary Files**

**File Name: Supplementary Data 1**

**Description:** Lipidomic analysis of sphingolipids in control, SMPDL3B knockdown (siSMP) and SMPDL3B overexpressing (SMP OE) podocytes, related to Figure 1.

**File Name: Supplementary Data 2**

**Description:** Illumina sequencing mRNA data analysis in SMPDL3b overexpressing podocytes compared to controls, related to Figure 2.

**File Name: Supplementary Data 3**

**Description:** Pathways enrichment analysis performed using DAVID 6.8. database in SMPDL3B overexpressing human podocytes, related to Figure 2.

**File Name: Supplementary Data 4**

**Description:** Lipidomic analysis of the sphingolipid content in kidney cortexes of control and podocyte-specific Smpdl3b deficient mice, related to Figure 4.

**File Name: Supplementary Data 5**

**Description:** Lipidomic analysis of sphingolipids in kidney cortexes of control and type 2 diabetic podocyte-specific Smpdl3b deficient mice, related to Figure 5.
