# Supplementary material for: SMPDL3b modulates insulin receptor signaling in diabetic kidney disease
Source: Nat Commun. 2019 Jun 19;10:2692. doi: 10.1038/s41467-019-10584-4 (PMC6584700; doi:10.1038/s41467-019-10584-4)
Supplement: Supplementary file 9 — Reporting Summary [file 41467_2019_10584_MOESM9_ESM.pdf]

nature research

Corresponding author(s): Alessia Fornoni

Last updated by author(s): Apr 20, 2019

Reporting Summary

Nature Research wishes to improve the reproducibility of the work that we publish. This form provides structure for consistency and transparency in reporting. For further information on Nature Research policies, see Authors & Referees and the Editorial Policy Checklist.

Statistics

For all statistical analyses, confirm that the following items are present in the figure legend, table legend, main text, or Methods section.

n/a	Confirmed	
☐	☒	The exact sample size (*n*) for each experimental group/condition, given as a discrete number and unit of measurement
☐	☒	A statement on whether measurements were taken from distinct samples or whether the same sample was measured repeatedly
☐	☒	The statistical test(s) used AND whether they are one- or two-sided<br>*Only common tests should be described solely by name; describe more complex techniques in the Methods section.*
☒	☐	A description of all covariates tested
☒	☐	A description of any assumptions or corrections, such as tests of normality and adjustment for multiple comparisons
☐	☒	A full description of the statistical parameters including central tendency (e.g. means) or other basic estimates (e.g. regression coefficient) AND variation (e.g. standard deviation) or associated estimates of uncertainty (e.g. confidence intervals)
☐	☒	For null hypothesis testing, the test statistic (e.g. *F*, *t*, *r*) with confidence intervals, effect sizes, degrees of freedom and *P* value noted<br>*Give P values as exact values whenever suitable.*
☒	☐	For Bayesian analysis, information on the choice of priors and Markov chain Monte Carlo settings
☒	☐	For hierarchical and complex designs, identification of the appropriate level for tests and full reporting of outcomes
☐	☒	Estimates of effect sizes (e.g. Cohen's *d*, Pearson's *r*), indicating how they were calculated

Our web collection on statistics for biologists contains articles on many of the points above.

Software and code

Policy information about availability of computer code

Data collection	No custom algorithms or software were used.
Data analysis	No custom algorithms or software were used.

For manuscripts utilizing custom algorithms or software that are central to the research but not yet described in published literature, software must be made available to editors/reviewers. We strongly encourage code deposition in a community repository (e.g. GitHub). See the Nature Research guidelines for submitting code & software for further information.

Data

Policy information about availability of data

All manuscripts must include a data availability statement. This statement should provide the following information, where applicable:

- Accession codes, unique identifiers, or web links for publicly available datasets
- A list of figures that have associated raw data
- A description of any restrictions on data availability

The raw data of the lipidomic analysis of human podocytes and kidney cortexes of podocyte-specific Smpdl3b knockout mice and podocyte-specific Smpdl3b deficient type 2 diabetic mice are available in the Supplementary Data 1 (related to Fig. 1), Supplementary Data 4 (related to Fig. 4) and Supplementary Data 5 (elated to Fig. 5), respectively. All sequencing data that support the findings of this study are available in the Supplementary Data 2 and Supplementary Data 3 (both related to Fig. 2) and have been deposited in the National Center for Biotechnology Information Gene Expression Omnibus (GEO) and are accessible through the GEO Series accession number GSE129666.

Field-specific reporting

Please select the one below that is the best fit for your research. If you are not sure, read the appropriate sections before making your selection.

☒ Life sciences ☐ Behavioural & social sciences ☐ Ecological, evolutionary & environmental sciences

For a reference copy of the document with all sections, see nature.com/documents/nr-reporting-summary-flat.pdf

Life sciences study design

All studies must disclose on these points even when the disclosure is negative.

Sample size	Minimal group sizes for in vitro and in vivo studies were determined via power calculation using the DSS Researcher's Toolkit with an α of 0.05.
Data exclusions	Graph Prism Outlier calculator software (https://www.graphpad.com/quickcalcs/Grubbs1.cfm) was used to indicate outliers in each set of data obtained for in vitro and in vivo experiments. Significant outliers were excluded from further statistical analysis.
Replication	Cell culture samples and mice were allocated randomly.
Randomization	Animals were grouped unblinded, but randomized. Three to five biological replicates were used for in vitro experiments on human podocytes or HEK293 cells.
Blinding	Histology analysis, including PAS, H&E, TEM, IHC, and genotyping of newly generated podocyte-specific Smpdl3b deficient mice were performed by two blinded independent investigators.

Reporting for specific materials, systems and methods

We require information from authors about some types of materials, experimental systems and methods used in many studies. Here, indicate whether each material, system or method listed is relevant to your study. If you are not sure if a list item applies to your research, read the appropriate section before selecting a response.

Materials & experimental systems

n/a	Involved in the study	
☐	☒	Antibodies
☐	☒	Eukaryotic cell lines
☒	☐	Palaeontology
☐	☒	Animals and other organisms
☒	☐	Human research participants
☒	☐	Clinical data

Methods

n/a	Involved in the study	
☒	☐	ChIP-seq
☐	☒	Flow cytometry
☒	☐	MRI-based neuroimaging

Antibodies

Antibodies used	Anti-caveolin-1 (#3251 for phospho-Cav1(Tyr14), #3267 for Cav1 (D46G3) XPTM), anti-insulin receptor beta subunit (4B8) (#3025), anti-Na/K-ATPase (#3010), anti-MEK-1/2 (D1A5) (#8727), anti-AKT (#9271 for phospho-AKT (Ser473) (D9E), #9272 for tAKT), anti-p70S6 kinase (#9234 for phosphor-p70S6K (Thr389 (108D2)), #9202 for p70S6K), anti-4EBP1 (#2855 for phospho-4E-BP1 (Thr37/46) (236B4), #9644 for 4E-BP1) primary antibodies were purchased from Cell Signaling Technology (MA, USA). SMPDL3B antibodies purchased from GenWay Biotech (CA, USA, #GWB-2281D4). Insulin receptor beta subunit antibodies (CT-1) for endogenous IP (#MA5-13778) were obtained from ThermoFisher Scientific Inc., HRP-conjugated secondary antibodies from Promega Corp. (WI, USA), anti-rabbit TrueBlot HRP-conjugated secondary antibodies from Rockland (PA, USA, #18-8816-31). For immunofluorescence primary rabbit polyclonal anti-Wilms' tumor 1 (WT1; #sc-192) and anti-goat polyclonal synaptopodin (P19; #sc-21537) antibodies were purchased from Santa Cruz Biotechnology (TX, USA), rabbit polyclonal SMPDL3b antibodies from GenWay (#GWB-2281D4) and rabbit monoclonal pAKT (Ser473; #9271) antibodies from Cell Signaling Technology. Alexa fluorescence 568 (red; #A10042; #11061), 488 (green; # A11059) secondary antibodies and DAPI (#D1306) were obtained from Invitrogen | Thermo Fisher Scientific (CA, USA). For Western Blot, a 1:1,000 dilution for primary antibodies was used and a 1:10,000 dilution for secondary antibodies was used. For flow cytometry, Human/Mouse Insulin R/CD220 APC-conjugated antibodies (#FAB1544A) and isotope control IgG APC-conjugated antibodies (#IC108A) were obtained from R&D Systems.
Validation	- phospho-Caveolin-1 (Tyr14): polyclonal anti-rabbit; human, mouse, rat, simian cross-reactivity. Provider validation statement including relevant citations is available here https://www.cellsignal.com/products/primary-antibodies/phospho-caveolin-1-tyr14-antibody/3251. Antibodypedia database profile is available here https://www.antibodypedia.com/gene/3530/CAV1/antibody/106395/3251.<br>- Caveolin-1 (D46G3): monoclonal anti-rabbit; human, mouse, rat, hamster, monkey, bovine, dog cross-reactivity. Provider validation statement including relevant citations is available here https://www.cellsignal.com/products/primary-antibodies/caveolin-1-d46g3-xp-rabbit-mab/3267. Antibodypedia database profile is available here https://www.antibodypedia.com/

gene/3530/CAV1/antibody/106409/3267.
- Insulin receptor beta (4B8): monoclonal anti-rabbit; human, mouse, rat cross-reactivity. Provider validation statement including relevant citations is available here https://www.cellsignal.com/products/primary-antibodies/insulin-receptor-b-4b8-rabbit-mab/3025. Antibodypedia database profile is available here https://www.antibodypedia.com/gene/3403/INSR/antibody/106233/3025.
- Na,K-ATPase: polyclonal anti-rabbit; human, mouse, rat, hamster, simian cross-reactivity. Provider validation statement including relevant citations is available here https://www.cellsignal.com/products/primary-antibodies/na-k-atpase-antibody/3010. Antibodypedia database profile is available here https://www.antibodypedia.com/gene/4542/ATP1A1/antibody/106224/3010.
- MEK1/2 (D1A5): monoclonal anti-rabbit; human, mouse, rat, monkey, D. melanogaster cross-reactivity. Provider validation statement including relevant citations is available here https://www.cellsignal.com/products/primary-antibodies/mek1-2-d1a5-rabbit-mab/8727?site-search-type=Products&N=4294956287&Ntt=mek1%2F2+&fromPage=plp.
- Phospho-AKT (Ser473): polyclonal anti-rabbit; Human, Mouse, Rat, Hamster, D. melanogaster, Bovine, Dog, Pig cross-reactivity. Provider validation statement including relevant citations is available here https://www.cellsignal.com/products/primary-antibodies/phospho-akt-ser473-antibody/9271.
- AKT: polyclonal anti-rabbit; Human, Mouse, Rat, Hamster, Monkey, Chicken, D. melanogaster, Bovine, Dog, Pig, Guinea Pig cross-reactivity. Provider validation statement including relevant citations is available here https://www.cellsignal.com/products/primary-antibodies/akt-antibody/9272.
- Phospho-p70 S6 Kinase (Thr389) (108D2): monoclonal anti-rabbit; Human, Mouse, Rat, Monkey cross-reactivity. Provider validation statement including relevant citations is available here https://www.cellsignal.com/products/primary-antibodies/phospho-p70-s6-kinase-thr389-108d2-rabbit-mab/9234.
- p70 S6 Kinase: polyclonal anti-rabbit; Human, Mouse, Rat, Monkey cross-reactivity. Provider validation statement including relevant citations is available here https://www.cellsignal.com/products/primary-antibodies/p70-s6-kinase-antibody/9202. Antibodypedia database profile is available here https://www.antibodypedia.com/gene/3544/RPS6KB1/antibody/107864/9202.
- SMPDL3b: polyclonal anti-rabbit; Bovine, Dog, Guinea pig, Horse, Human, Mouse, Pig, Rabbit, Rat cross-reactivity. Provider validation statement is available here https://www.genwaybio.com/smpdl3b-sphingomyelin-phosphodiesterase-acid-like-3b. Relevant citations: 1) Fornoni et al. Sci Transl Med, 2011 https://www.ncbi.nlm.nih.gov/pubmed/21632984; 2) Yoo TH et al., J Am Soc Nephrol, 2015 https://www.ncbi.nlm.nih.gov/pubmed/24925721; 3) Ahmad A et al., FASEB J, 2017 https://www.ncbi.nlm.nih.gov/pubmed/27836988.
- Phospho-4E-BP1 (Thr37/46) (236B4): monoclonal anti-rabbit; Human, Mouse, Rat, Monkey, D. melanogaster cross-reactivity. Provider validation statement including relevant citations is available here https://www.cellsignal.com/products/primary-antibodies/phospho-4e-bp1-thr37-46-236b4-rabbit-mab/2855. Antibodypedia database profile is available here https://www.antibodypedia.com/gene/3558/EIF4EBP1/antibody/106120/2855.
- 4E-BP1 (53H11): monoclonal anti-rabbit; Human, Mouse, Rat, Monkey cross-reactivity. Provider validation statement including relevant citations is available here https://www.cellsignal.com/products/primary-antibodies/4e-bp1-53h11-rabbit-mab/9644. Antibodypedia database profile is available here https://www.antibodypedia.com/gene/3558/EIF4EBP1/antibody/108060/9644.
- Insulin receptor beta subunit: monoclonal anti-rabbit; Human, Mouse, Rat cross-reactivity. Provider validation statement including relevant citations is available here https://www.thermofisher.com/antibody/product/INSR-beta-Antibody-clone-CT-1-Monoclonal/MA5-13778.

Eukaryotic cell lines

Policy information about cell lines

Cell line source(s)	Normal human podocytes (gift from Dr. Jochen Reiser, Rush University, Chicago), HEK293 cells (ATCC), SMPDL3b overexpression human podocytes (developed by Fornoni et al., Sci Transl Med, 2011).
Authentication	Immortalized human podocytes are regularly checked for podocyte like morphology by phalloidin staining and podocyte marker protein expression such as synaptopodin and podocin by Western blot. Stable podocyte cell line expressing GFP-labeled SMPDL3b was developed by electroporation (MammoZapper cloning gun, Tritech Research) as reported previously (Fornoni et al., Sci Transl Med, 2011).<br>Plasmid constructs were sequence verified before use. Knockdown or overexpression of the target gene was analyzed by quantitative real-time PCR and Western blot analysis using previously validated real-time PCR primers and commercially available antibodies, respectively was performed.<br>Some plasmids for cloning were obtained commercially. The identity of cloning plasmids was verified by sequence analysis. All remaining biological and chemical reagents encompassed by the proposed research protocols are commercially available and therefore underwent company-determined authentication procedures.
Mycoplasma contamination	All cell lines used tested were negative for mycoplasma contamination.
Commonly misidentified lines<br>(See ICLAC register)	No commonly misidentified cell lines were used in the current study.

Animals and other organisms

Policy information about studies involving animals; ARRIVE guidelines recommended for reporting animal research

Laboratory animals	- Mice in which exon 2 of Smpdl3b is flanked by loxP sites were purchased from the International Knockout Mouse Consortium (B6N;B6N-SMPDL3btm1a(EUCOMM)Wtsi/H; #MGI:1916022); females and males; 4-6 w.o.<br>- Mice carrying a Flp-recombinase transgene (B6.129S4-Gt(ROSA)26Sortm1(FLP1)Dym/RainJ; #009086) were purchased from the Jackson Laboratories (USA); females and males; 4-6 w.o.<br>- mice carrying a Cre-recombinase transgene specifically expressed in podocytes (B6.Cg-Tg(NPHS2-cre)295Lbh/J; catalog #008205) were purchased from the Jackson Laboratories (USA); females and males; 4-6 w.o.<br>- Leprdb heterozygous db/+ and homozygous db/db mice (B6.BKS(D)-Lepr<db>/J; catalog #000697) were purchased from the

	Jackson Laboratories (USA); females; 4 w.o. for breeding (related to db/+ only) and 10 w.o. for exogenous C1P treatment.<br>- Podocyte-specific Smpdl3b deficient mice were produced in the laboratory and validated for the first time in the current study. Twenty eight week old females and males were used in the study.<br>All mice were authenticated and identified by established genotyping methods using standard PCR on tail biopsies. Expression levels in target organs was determined using commercially available reagents.
Wild animals	The study did not involved wild animals.
Field-collected samples	The study did not involved samples collected form the field.
Ethics oversight	All animal studies have complied with all relevant ethical regulations and were performed in accordance with the National Institutes of Health Guidelines. The study protocol was approved by the Institutional Animal Care and Use Committee of the University of Miami, Miller School of Medicine.

Note that full information on the approval of the study protocol must also be provided in the manuscript.

Flow Cytometry

Plots

Confirm that:

☒ The axis labels state the marker and fluorochrome used (e.g. CD4-FITC).

☒ The axis scales are clearly visible. Include numbers along axes only for bottom left plot of group (a 'group' is an analysis of identical markers).

☒ All plots are contour plots with outliers or pseudocolor plots.

☒ A numerical value for number of cells or percentage (with statistics) is provided.

Methodology

Sample preparation	CTRL or SMP OE human podocytes were collected at day 14 of differentiation and incubated with 0.5% BSA, 0.1% Saponin in 1xPBS for 10 min at room temperature followed by Fc blocking step for 10 min at room temperature using 12.5 µg of human BD Fc Block solution. Incubation with 30 µl of insulin receptor APC-conjugated antibodies per sample was performed for 1h at room temperature in dark. HEK293 insulin receptor transfected cells were served as positive control. CTRL human podocytes incubated with 10 µl of isotop control IgG APC-conjugated antibodies were used as a negative control.
Instrument	Results were read on BD LSR II 15-color flow cytometric analyzer using PE fluorochrome with laser lens 535 nm and emission filters 585/15.
Software	BD FACSDiva 8.0.2 software were used to analyze the data obtained. Data were further analyzed with GraphPad Prism version 7.0.
Cell population abundance	Purity of post-sort fractions is measured by flow cytometry core facility and >90%.
Gating strategy	Gating strategies are provided in a Supplementary Information file.

☒ Tick this box to confirm that a figure exemplifying the gating strategy is provided in the Supplementary Information.
